# The Physical Signs of Enlargement of the Gall-Bladder

**Published:** 1910-11-26

**Authors:** 


### Medicine.

### THE PHYSICAL SIGNS OF ENLARGEMENT OF THE GALL-BLADDER.

A normal gall-bladder cannot, as a rule, be felt. A slight enlargement does not give rise to any visible local distension or prominence of the abdominal wall. If the enlargement is considerable, a distinct local prominence of the abdominal wall may be seen on the right side of the abdomen, usually below and in a line with the ninth costal cartilage. If the patient takes a deep breath, this local protuberance may be seen to move distinctly downwards beneath the abdominal wall.

The tumour if watched from day to day may be noticed to vary in size especially when it is associated with the presence of gall-stones. On palpation, a rounded, smooth, elastic, pyriform swelling may be felt, which varies in size from being just perceptible to the touch to being a tumour as large as an ovarian cyst; it is exceptional for it to be bigger than a large pear. The position of the tumour varies according to the size of the liver. If the liver is not enlarged the tumour is felt just below the ninth right costal cartilage. As the liver increases in size the enlarged gall-bladder, as a rule, passes towards the left. If associated with considerable enlargement of the liver it may be situated at the level of, or below the umbilicus, or even a little to the left of it. On bimanual palpation the tumour is felt to be superficial and not to extend backwards into the loin so as to fill out the flaccid space outside the erector spinæ muscle.

The upper end is narrowed and fixed to the under surface of the liver, the edge of which may or may not be detected by palpation. The larger end, which is below, is usually free and movable. It may be moved from side to side and from before backwards, but cannot be pulled downwards towards the pelvis and separated from the liver. If the tumour is pushed backwards towards the loin, as soon as the pressure on it is released it springs back to its original position close under the abdominal wall. A distinct groove may generally be felt between the upper part of the tumour and the liver. The mass may be felt to move downwards synchronously with the liver when the patient takes a deep breath. During expiration it moves upwards again, and even if grasped by both hands it cannot be retained in the position it assumes at the end of a deep inspiration.

If the enlargement is due to the presence of calculi and malignant disease it may give rise to a hard nodular tumour, and in the case of calculi a sensation of crackling may occasionally be obtained when it is manipulated. If the patient is placed in the knee-elbow position and the abdomen is palpated, an enlarged gall-bladder will be felt to be close against the abdominal wall and to rest, as it were, on the hand. If the gall-bladder is large from distension with fluid, a thrill may be obtained on flicking the tumour as in testing for hydatid fremitus. Dulness on percussion may or may not be present, depending a good deal on the degree of distension of the adjacent coils of intestine. If there has been inflammation leading to local peritonitis followed by matting together of the adjacent viscera the tumour may be fixed, immovable, and tender, and give rise to a distinctly dull note on percussion. On percussing the loin the normal resonant note due to the colon in this situation is present.

Jaundice may or may not be associated. If the distension is due to obstruction of the common bile-duct icterus is usually present. A distended gall-bladder may contain pus, mucus, or bile. An exploratory laparotomy may be required, therefore, in a doubtful case to distinguish it from hydatid cyst, hydronephrosis, or ovarian tumour.

